# The Munduruku marmoset: a new monkey species from southern Amazonia

**DOI:** 10.7717/peerj.7019

**Published:** 2019-07-25

**Authors:** Rodrigo Costa-Araújo, Fabiano R. de Melo, Gustavo Rodrigues Canale, Sandra M. Hernández-Rangel, Mariluce Rezende Messias, Rogério Vieira Rossi, Felipe E. Silva, Maria Nazareth Ferreira da Silva, Stephen D. Nash, Jean P. Boubli, Izeni Pires Farias, Tomas Hrbek

**Affiliations:** 1 Pós-graduação em Ecologia, Instituto Nacional de Pesquisas da Amazônia, Manaus, Amazonas, Brasil; 2 Departamento de Genética, Universidade Federal do Amazonas, Manaus, Amazonas, Brasil; 3 Departamento de Engenharia Florestal, Universidade Federal de Viçosa, Viçosa, Minas Gerais, Brasil; 4 Unidade Acadêmica Especial Ciências Biológicas, Universidade Federal de Goiás, Jataí, Goiás, Brasil; 5 ICNHS/CUS/NEBAM, Universidade Federal de Mato Grosso, Sinop, Mato Grosso, Brasil; 6 Departamento de Biologia, Universidade Federal de Rondônia, Porto Velho, Rondônia, Brasil; 7 Departamento de Biologia e Zoologia, Instituto de Biociências, Universidade Federal de Mato Grosso, Cuiabá, Mato Grosso, Brasil; 8 School of Environment and Life Sciences, University of Salford, Salford, Manchester, UK; 9 Ecovert, Instituto de Desenvolvimento Sustentável Mamirauá, Tefé, Amazonas, Brasil; 10 Coleção de Mamíferos, Instituto Nacional de Pesquisas da Amazônia, Manaus, Amazonas, Brasil; 11 Department of Anatomical Sciences, Stony Brook University, Stony Brook, NY, USA

**Keywords:** Species discovery, Integrative taxonomy, Field exploration, Arc of deforestation, Jamanxim River, Tapajós River

## Abstract

Although the Atlantic Forest marmosets (*Callithrix* spp.) are among the best studied Neotropical primates, the Amazonian marmosets (*Callibella humilis, Cebuella* spp. and *Mico* spp.) are much less well-known. Even species diversity and distributions are yet to be properly determined because field data and materials currently available in scientific collections do not allow comprehensive taxonomic studies of Amazonian marmosets. From 2015 to 2018, we conducted 10 expeditions in key-areas within southern Amazonia where little or no information on marmosets was available. In one such region—the Tapajós–Jamanxim interfluve—we recorded marmosets with a distinctive pelage pigmentation pattern suggesting they could represent a new species. We tested this hypothesis using an integrative taxonomic framework that included phylogenomic data (ddRAD sequences), pelage pigmentation characters, and distribution records. We found that the marmosets of the northern Tapajós–Jamanxim interfluve have unique states in pelage pigmentation characters, form a clade (100% support) in our Bayesian and Maximum-Likelihood phylogenies, and occur in an area isolated from other taxa by rivers. The integration of these lines of evidence leads us to describe a new marmoset species in the genus *Mico*, named after the Munduruku Amerindians of the Tapajós–Jamanxim interfluve, southwest of Pará State, Brazil.

## Introduction

The marmosets, or saguis, are small Neotropical primates. Marmosets have claw-like nails on all digits except the hallux, used for the vertical climbing of and leaping between trees, as well as long lower jaw incisors and incisor-like canines used to gouge holes in the bark of trees so that they can obtain tree exudates, an important component of their diets (Coimbra-Filho & Mittermeier, 1976; Hershkovitz, 1977; Rosenberger, 1980). The marmosets are currently classified in four genera of the family Callitrichidae—*Callibella*, *Callithrix*, *Cebuella* and *Mico*: the species of genera *Mico* and *Callibella* are found in south-eastern Amazonia, *Cebuella* taxa in south-western and north-western Amazonia, and *Callithrix* species in the Atlantic Forest, Cerrado, and Caatinga biomes (Rylands, Coimbra-Filho & Mittermeier, 2009; Rylands & Mittermeier, 2013; Silva et al., 2018).

Currently, species diversity and distributions of Amazonian marmosets are poorly known due to scarcity of basic field data, museum material and lack of samples suitable for molecular analyses. Most of the material available on Amazonian marmosets stored in museums was obtained in the 18th and 19th centuries by naturalists and professional collectors venturing into southern Amazonia, with few additional specimens being collected in the last three decades. Thus, specimens available for study are restricted to few localities and biological samples are nearly absent from tissue collections. There are many areas within and between known species distributions of *Mico* that most likely harbor marmosets although these areas are little explored or never have been surveyed. Consequently, reliable taxonomic assessment using either morphological or molecular data is hampered for most of the Amazonian marmosets.

The Tapajós–Jamanxim interfluve, in the southwest of Pará State, southern Brazilian Amazonia, is a poorly studied region and the taxonomic identity of marmosets that occur there is uncertain. The Tapajós–Jamanxim interfluve encompasses the forests west of the Jamanxim River and east of the upper Tapajós River and east of the lower Teles Pires River, including the basins of the Crepori, Cururú, São Benedito, and Novo Rivers. The distribution map of *Mico* species presented in the last comprehensive literature review shows no record in the Tapajós–Jamanxim interfluve (Rylands, Coimbra-Filho & Mittermeier, 2009). Fialho (2010) presented a record of *Mico leucippe* Thomas, 1922 on the right margin of São Benedito River, in the southern portion of the Tapajós–Jamanxim interfluve. More recently, two field surveys (Ravetta, 2015; Buss et al., 2017) reported the occurrence of *M. leucippe* from three areas in the northern portion of Tapajós–Jamanxim interfluve, on the left margin of the middle and lower section of the Jamanxim River. However, no reference material from this region was available, whether voucher specimens or tissue samples, and thus no taxonomic analysis was conducted with the Tapajós–Jamanxim marmosets until this study.

Recently, we conducted field surveys in the forests of the Tapajós–Jamanxim interfluve to investigate the taxonomy of marmosets in this region. During our surveys, marmosets bearing a pattern of pelage pigmentation distinctive from *M. leucippe* and other *Mico* species were observed in the northern portion of Tapajós–Jamanxim interfluve, suggesting that these individuals could belong to a hitherto unknown species. To test this hypothesis, we used the integrative protocol proposed by Schlick-Steiner et al. (2010) for taxonomic assessment. We collected and analyzed genomic data of multiple species of *Mico*, data of pelage pigmentation characters traditionally considered important for the delimitation of primate species, and distribution records. The integrative approach in taxonomy provides an objective framework to test species hypotheses and can support more stable species-names and classifications (Padial et al., 2010). Specifically, the use of phenotypic/morphological data associated with nuclear DNA sequence data is preferable: combined, they contribute to a greater accuracy in taxonomic decisions and accommodate practical constraints of data collection and analyses (Schlick-Steiner et al., 2010). Using this integrated taxonomic approach, we describe a new species of *Mico* from the northern Tapajós–Jamanxim interfluve.

## Methods

### Fieldwork

We conducted marmoset surveys in southern Amazonia (east of the Madeira and south of the Amazonas Rivers) during 10 field expeditions between May 2015 and April 2018, four of which included the Tapajós–Jamanxim interfluve. Our surveys consisted of trekking in the forest or canoeing up streams while playing long-call recordings of *M. argentatus* (Emmons, Whitney & Roos, 1998) and *M. marcai* (R. Costa-Araújo, 2015, personal data) to stimulate vocalization responses of marmosets. For this, a custom MP3 player was connected to a Marshall MS-2 mini amplifier. For each observation, we recorded the geographic coordinates, the time, and the type of habitat in which each individual or troop was observed. To allow the study of marmosets bearing distinctive pelage pigmentation we collected specimens and samples of muscular tissue in the field, under a permit (n° 50416) provided by the Instituto Chico Mendes de Conservação da Biodiversidade (ICMBio) and following the established guidelines for studies of primates in protected areas of Amazonia (Vidal, 2012). The collected specimens were prepared and deposited in the mammal collections of the Instituto Nacional de Pesquisas da Amazônia (INPA) and Museu Paraense Emilio Goeldi (MPEG), and the tissue samples were preserved in 96% ethanol and deposited in the Coleção de Tecidos de Genética Animal (CTGA) of the Universidade Federal do Amazonas.

### Criteria-driven test of species hypothesis using an integrative approach

Based on our field observations we hypothesized that the marmosets of the northern Tapajós–Jamanxim interfluve represent a species new to science. We tested this hypothesis within an integrative taxonomic framework, incorporating data from pelage pigmentation characters, phylogenetic relationships inferred from genomic DNA sequences, and geographic distribution. All sources of information were analyzed separately and then synthesized for decision making (Table S1) following Schlick-Steiner et al. (2010).

### Pelage pigmentation data

We collected data of qualitative characters of pelage pigmentation in chromogenetic fields, following Hershkovitz (1977). Chromogenetic fields are certain parts of a mammalian body that accumulate more variation in pelage pigmentation and thus provide a useful basis for taxonomic studies (Hershkovitz, 1968, 1977). Variation in pelage pigmentation in chromogenetic fields have been used as a primary—and often the unique—source of information for taxonomic studies of Neotropical primate species and for descriptions of marmoset species (Hershkovitz, 1977; Vivo, 1991; Mittermeier, Schwarz & Ayres, 1992; Silva-Júnior & Noronha, 1998). We obtained data on pelage pigmentation patterns in 10 chromogenetic fields (Fig. S1) from 521 specimens representing all currently recognized species of *Callibella* and *Mico* through direct examination of prepared skins in the following mammalian collections: Field Museum of Natural History, INPA, MPEG, Museu de Zoologia da Universidade de São Paulo, Natural History Museum of London, Naturhistorisches Museum Wien, and Universidade Federal de Rondônia (Table S2). Additionally, we examined the pigmentation of the tegument of face and ears, and the size, quantity and insertion of ear-hair in all specimens. Our analysis here was based on comparisons of the states of pelage pigmentation in the 10 chromogenetic fields of marmosets from the Tapajós–Jamanxim interfluve (*n* = 6) and specimens from species closely related in terms of geographic distribution, pelage pigmentation patterns, and phylogenetic relationships: *M. argentatus* Linnaeus, 1771 (*n* = 150), *M. emiliae* Thomas, 1920 (*n* = 10), *M. intermedius* Hershkovitz, 1977 (*n* = 9), and *M. leucippe* (*n* = 28). In order to assess whether the marmosets from the northern Tapajós–Jamanxim interfluve are a new species we tested for diagnosability and geographic cohesion of the states of pelage pigmentation characters.

### Phylogenomic data

Our phylogenomic data consisted of a genome-wide sampling of nuclear DNA (Peterson et al., 2012). The total DNA was extracted from fresh tissue samples preserved in alcohol following Sambrook, Fritsch & Maniatis (1989), and processed to obtain thousands of loci per individual, each ranging from 320 to 400 base pairs (see https://github.com/legalLab). Based on phylogenetic relationships between some of *Mico* species (Sena et al., 2002; Schneider et al., 2012; Garbino, 2015; Silva et al., 2018), key synapomorphies found in tegument pigmentation on face and ears and in ear-hair size, quantity and insertion, and geographic distribution, we expect to find the species of the genus *Mico* grouped into three principal lineages. One lineage comprising *M. argentatus*, *M. emiliae*, and *M. leucippe*, a second lineage comprising *M. humeralifer* and *M mauesi*, and a third lineage comprising *M. marcai* and *M. melanurus*. We expected that marmosets of the northern Tapajós–Jamanxim interfluve should belong to the lineage of *M. argentatus*, *M. emiliae*, and *M. leucippe* because all these marmosets present reddish and almost naked tegument in face and ears, and occur in the Tapajós–Xingu interfluve. Accordingly, we sampled multiple individuals of the distinctive Tapajós–Jamanxim marmosets (*n* = 4), *M. argentatus* (*n* = 3), *M. emiliae* (*n* = 3) and *M. leucippe* (*n* = 3)—as well as *M. rondoni* (*n* = 1) and *M. intermedius* (*n* = 2), species of unknown phylogenetic relationships but sharing similarities of tegument and hair patterns on face and ears with the other four taxa. To root this ingroup and to propose preliminary phylogenomic hypothesis for Amazonian marmosets, we included representatives of the two additional lineages: *M. humeralifer* (*n* = 1) and *M. mauesi* (*n* = 1), and *M. marcai* (*n* = 1) and *M. melanurus* (*n* = 1); we also included *M. saterei* (*n* = 1) to clarify its phylogenetic relationship. The final data matrix consisted of concatenated loci with no more than 50% missing data per locus. *Callibella humilis* (*n* = 1), *Cebuella* cf. *niveiventris* (*n* = 1) and *Callithrix jacchus* (*n* = 1) were used as outgroups. In total 24 specimens of 14 marmoset taxa were sampled for genomic data (Table S3). The phylogenetic relationships were inferred using maximum likelihood in RAxML (Stamatakis, 2014) and Bayesian inference in ExaBayes (Aberer, Kobert & Stamatakis, 2014) under the GTR+gamma model of molecular evolution (Tavaré, 1986). Phylogenomic sequence alignment is available at the github repository (https://github.com/legalLab/publications).

## Results

### Pelage pigmentation data

**Figure 1:**
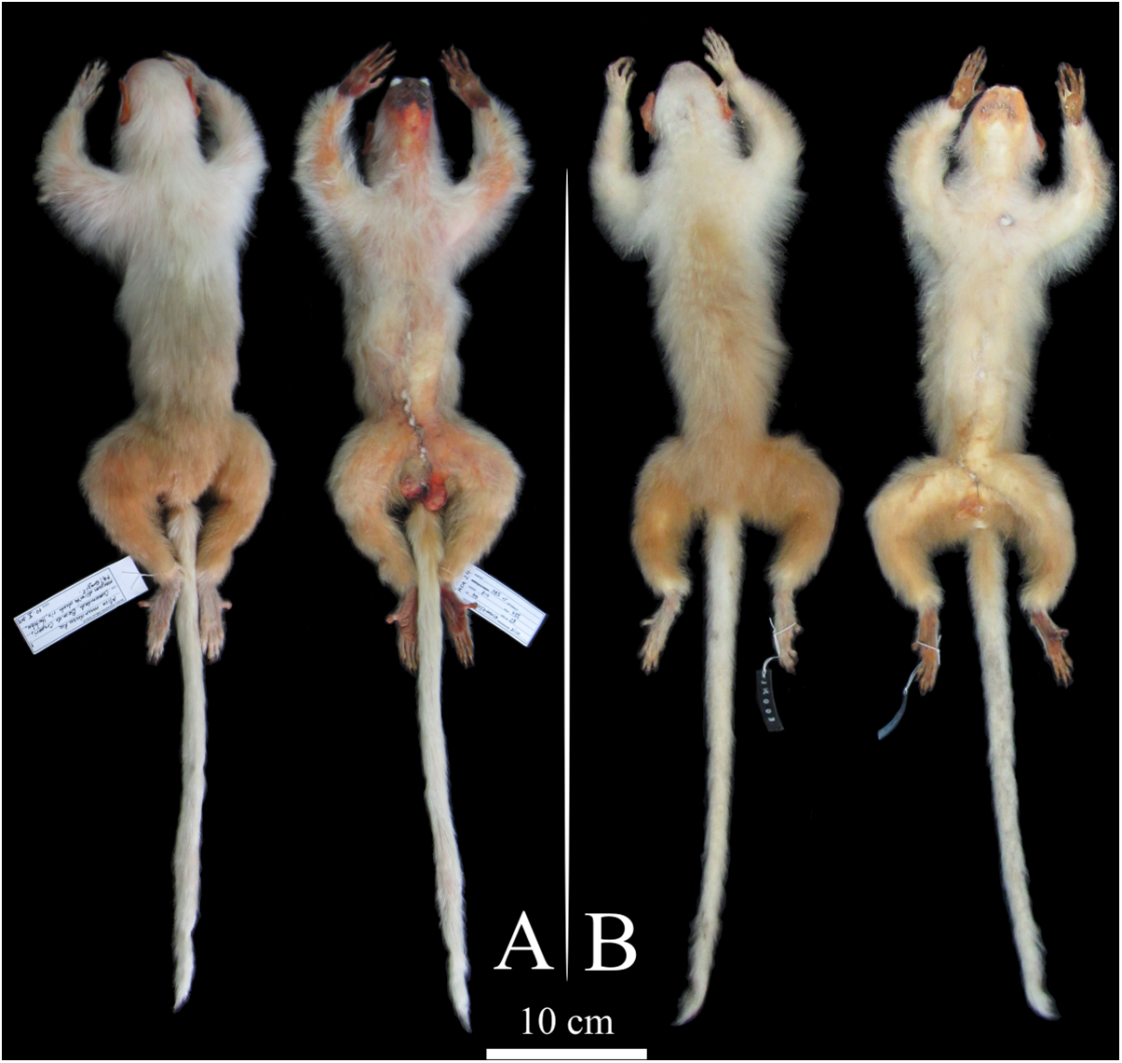
Dorsal and ventral views of *Mico munduruku* sp. n. (A) Holotype (MPEG 45560); (B) paratype (MPEG 45622).

All the marmosets from the northern Tapajós–Jamanxim interfluve show diagnostic states in pelage pigmentation characters. White tail, beige-yellowish saddle, white forearms with a beige-yellowish spot on the elbow, and white feet and hands (Fig. 1) are autapomorphies that readily distinguish these individuals from other species geographically proximate, similar in pelage pigmentation patterns, and phylogenetically closely related (Table 1; Fig. S2). Only minor tonal variation was observed in the saddle and forearm, clearly attributable to individual variation since it was observed within individuals of the same troop. In addition to the specimens we collected and recorded in the field, the MPEG has a specimen (MPEG 45622) (see Table S2) from the northern Tapajós–Jamanxim interfluve, which exhibits the same diagnostic characters described above. Body size and weight of marmosets from the Tapajós–Jamanxim interfluve is listed in Table 2.

**Table 1:** Pelage pigmentation characters (chromogenetic fields) and their states in *Mico munduruku* sp. n. and species closely related in terms of geographic distribution, pelage pigmentation patterns, and phylogenetic relationships.

Species/characters	*Mico munduruku* sp. n.	*Mico argentatus*	*Mico emiliae*	*Mico intermedius*	*Mico leucippe*
I. Crown	White	White	Black	Pale brown stripe	Cream
II. Head	White	White	Black and white	Silvery	Cream
III. Mantle	White	Silvery	Light brownish gray	Cream and silvery	Cream
IV. Forearms	White, beige-yellowish patch	Silvery	Blackish gray	Yellowish	Cream and golden
V. Hands	White	Dark gray	Black	Yellowish	Golden
VI. Saddle	Beige-yellowish	Silvery	Light grayish brown	Cream and black	Cream
VII. Rump	Dark yellow	Silvery	Light grayish brown	Blackish ochre	Cream
VIII. Underparts	Dark yellow	Silvery	Brown agouti	Blackish ochre	Cream and golden
IX. Feet	White	Dark gray	Black	Ochre	Golden
X. Tail	White	Black	Black	Cream and black	Golden

**Table 2:** Body size measurements (in centimeters) and weight (in grams) of *Mico munduruku* sp. n. type specimens.

Specimen	Head and Body	Tail	Ear	Foot	Weight
MPEG 45559	27.60	30.70	2.90	6.50	405
MPEG 45560	27.50	31.00	2.80	7.00	435
MPEG 45622	18.00	33.00	2.50	6.00	300
INPA 7284	25.30	27.70	2.27	6.13	270
INPA 7285	24.30	29.20	2.51	6.27	330
INPA 7382	21.50	28.20	2.34	6.15	250
Average (±SD)	24.03 (±3.4)	29.97 (±1.8)	2.55 (±0.2)	6.34 (±0.3)	331.67 (±67.7)

### Phylogenomic data

**Figure 2:**
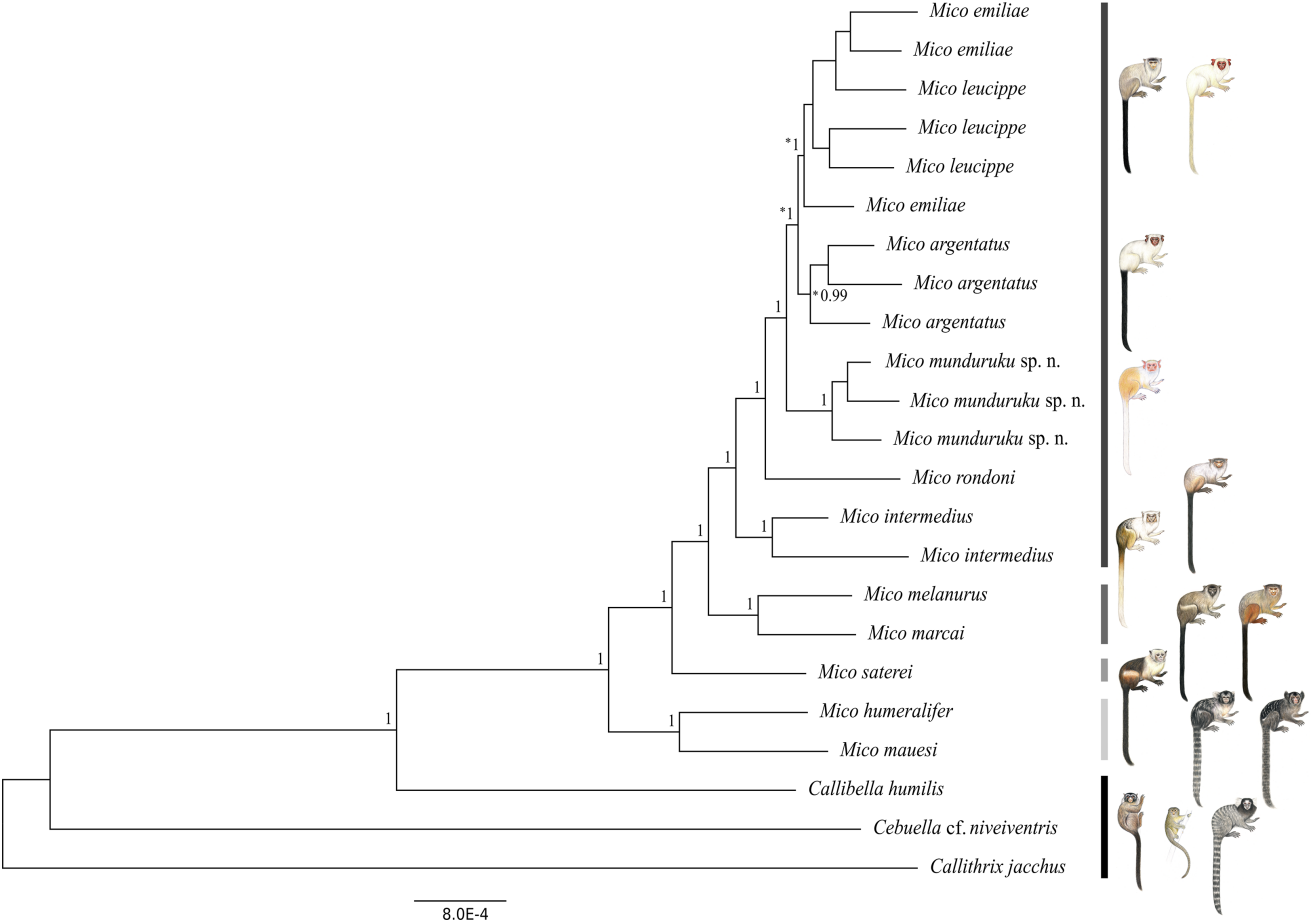
Bayesian phylogeny of the genus *Mico*. Gray-scale bars represent the main species lineages in genus *Mico*, black bar represent the outgroups. Clade posterior probabilities are given above nodes. Asterisk (*) indicates low (<70%) bootstrap support in the Maximum Likelihood phylogeny which otherwise was identical to the Bayesian inference phylogeny. Illustrations: Stephen Nash.

We obtained a genomic dataset consisting of 1,780 loci, 673,844 base pairs in total, each of 350 base pairs on average. Both Bayesian (Fig. 2) and Maximum Likelihood phylogenetic inference (Fig. S3) resulted in identical topologies. The specimens from the northern Tapajós–Jamanxim interfluve were recovered as a monophyletic group, sister to the other marmoset species of Tapajós–Xingu interfluve—*M. argentatus*, *M. emiliae* and *M. leucippe*; *M. emiliae*, and *M. leucippe* were recovered as polyphyletic in both phylogenies, sister to the *M. argentatus* clade; *M. rondoni* is sister to these four taxa from the Tapajós–Xingu interfluve and *M. intermedius* was recovered as sister to these five taxa, which form a lineage supported with pp = 1 and bp = 82%. *M. humeralifer* and *M. mauesi* were recovered as sister species (pp = 1; bp = 100%) and form a second main lineage of the genus *Mico*, as well as *M. marcai* and *M. melanurus*, sister species that represent a third main lineage of the genus (pp = 1; bp = 89%). *M. saterei* was retrieved as a fourth, single-species lineage (pp = 1; bp = 100%). *Callibella humilis* is a distinctive lineage sister to all *Mico* species analyzed here and strongly supported as such (pp = 1; bp = 100%). *Cebuella* cf. *niveiventris* is sister to both *Callibella humilis* and *Mico* taxa, and all these Amazonian marmoset genera are sister to *Callithrix jacchus*.

### Geographic distribution

**Figure 3:**
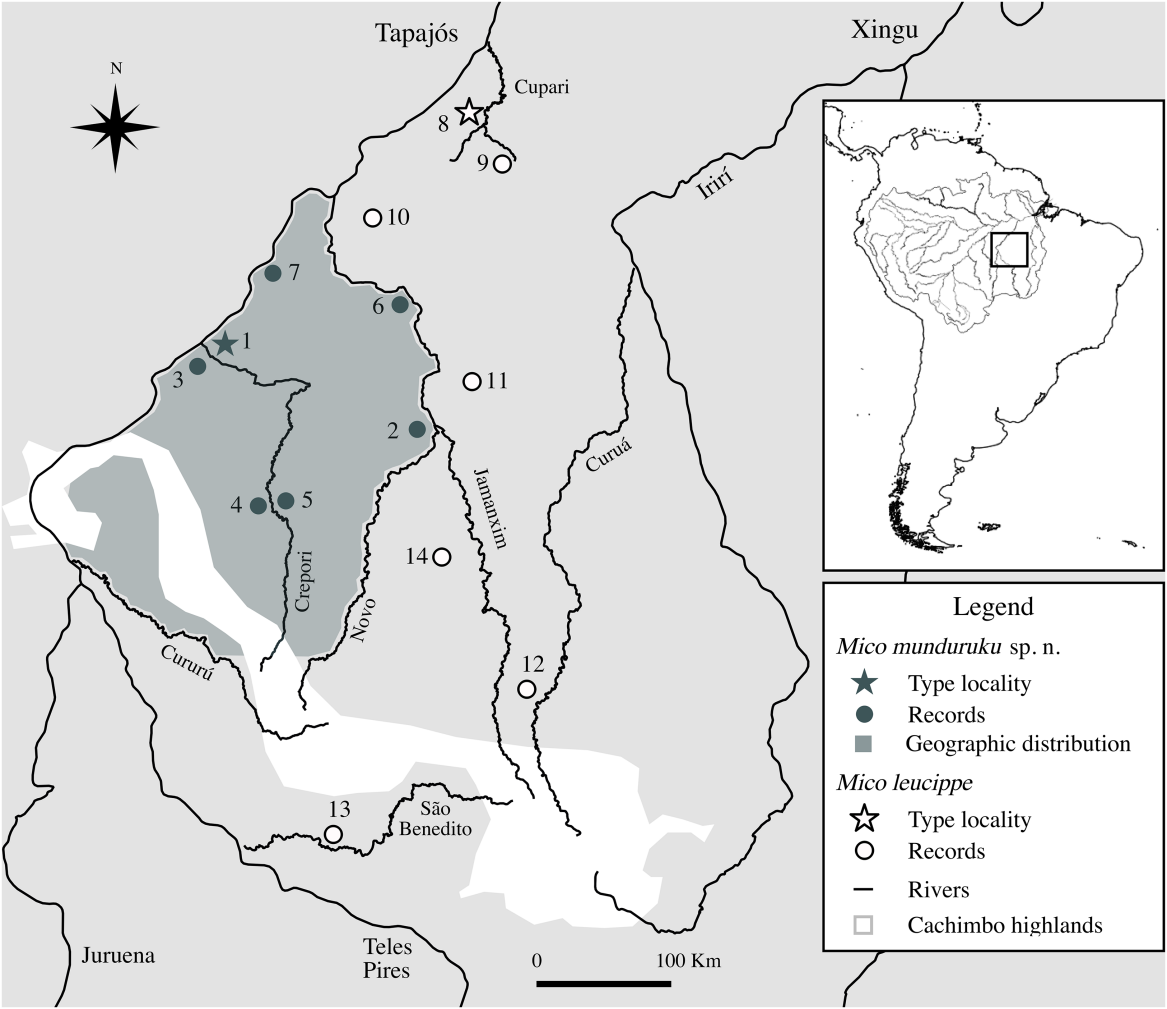
Geographic distribution of *Mico munduruku* sp. n. and records of *M. leucippe* in the Tapajós–Xingu interfluve, southern Amazonia (see Table 3 for locality details).

Based on specimens observed in the field, interviews with local inhabitants, specimens examined in museum collections and field reports, there are seven localities in the northern Tapajós–Jamanxim interfluve where the distinctive marmosets can be found (Fig. 3; Table 3), covering an area of approximately 55,000 km^2^. Based on these point occurrences, we hypothesize the geographic distribution of the distinctive marmosets is located in the northern portion of the Tapajós–Jamanxim interfluve, a region that is separated from other *Mico* species in the west and north by the Tapajós, in the east by the Jamanxim, and in the south by the Novo and Cururú Rivers. *M. leucippe* was also recorded in the Tapajós–Jamanxim interfluve, but only south of the Cururú and south and east of Novo Rivers, without evidence of sympatry with the distinctive marmosets (found to the north of these rivers). The matrix of dry vegetation and mountains of the Cachimbo highlands may act as an additional ecological/geographic barrier to both taxa in the headwaters of Novo and Cururú Rivers. Since previous records of marmosets in the northern Tapajós–Jamanxim interfluve were not based on direct specimen examination, but rather on visualization and/or vocalization recordings, we conclude that these were of the same distinctive species described below.

**Table 3:** Records considered for hypothesizing the geographic distribution of *Mico munduruku* sp. n. and *Mico leucippe* in southern Amazonia, Brazil.

ID	Locality	Municipality	State	Lat (DMS)	Long (DMS)	Type of record	Source	Specimen code
***Mico munduruku* sp. n.**								
1	Boca do Crepori community—type locality	Itaituba	PA	05°46′55″S05°45′59″S	57°15′14″W57°17′14″W	Preserved specimen	this study	MPEG 45559*^†^, 45560*, 45622^†^
2	Jardim do Ouro community, left margin of Jamanxim River	Itaituba	PA	06°15′11″S	55°46′49″W	Preserved specimen	this study	INPA 7284*^†^, 7285*, 7382*^†^
3	Jacu community	Jacareacanga	PA	05°47′02″S	57°21′18″W	Survey—interview	this study	–
4	Floresta Nacional do Crepori	Jacareacanga	PA	06°49′00″S	56°51′29″W	Survey—interview	this study	–
5	Creporizão community	Itaituba	PA	06°49′14″S	56°50′46″W	Survey—interview	this study	–
6	Jamanxim	Itaituba	PA	05°27′56″S	55°55′39″W	Survey	Ravetta (2015)	–
7	Ratão stream	Itaituba	PA	05°22′33″S	56°55′24″W	Survey	Ravetta (2015)	–
***Mico leucippe***								
8	Pimental, Rio Tapajós—type locality	Itaituba	PA	04°34′00″S	56°12′00″W	Preserved specimen	Thomas (1922)	NHM 1909.3.9.2
9	Cupari River, left margin	Rurópolis	PA	04°10′40″S	55°25′51″W	Preserved specimen	this study	MPEG 45567*, 45568*^†^, 45569*^†^
10	Trairão	Trairão	PA	04°53′05″S	56°10′46″W	Preserved specimen	this study	MPEG 45563*, 45564*, 45565*
11	Parque Nacional do Jamanxim	Itaituba	PA	05°58′14″S	55°41′40″W	Survey	Ravetta (2015)	–
12	Castelo dos Sonhos	Altamira	PA	08°02′51″S	55°08′39″W	Road-killed animal	this study	CTGA-M 5912*^†^
13	São Benedito River	Jacareacanga	PA	09°05′39″S	56°31′37″W	Survey; Preserved specimen	Fialho (2010); this study	MPEG 45569*
14	Nardino farm	Novo Progresso	PA	07°09′38″S	55°42′55″W	Survey	Ravetta (2015)	–

**Notes:**

Localities ID follow the locality numbers given in the distribution map presented here (Fig. 3), asterisks (^*^) and crosses (^†^) indicate specimens used for collection of pelage pigmentation and phylogenomic data, respectively.

INPA, Instituto Nacional de Pesquisas da Amazônia; MPEG, Museu Paraense Emilio Goeldi; NHM, Natural History Museum of London; CTGA-M, Coleção de Tecidos de Genética Animal.

The distinctive marmosets of the northern Tapajós–Jamanxim interfluve form a highly supported clade in our phylogeny of the genus *Mico*, share unique states in pelage pigmentation characters—which show abrupt transitions when compared to all species of *Mico* closely related in terms of pelage pigmentation, phylogenetic relationships and geographic distribution—and the geographic distribution of these marmosets is parapatric with respect to *M. leucippe* and allopatric with respect to *M. argentatus*, *M. emiliae* and other taxa of *Mico*. Each of these distinct lines of evidence lead us to reject the hypothesis that the marmosets of the northern portion of Tapajós–Jamanxim interfluve belong to *M. leucippe* or other already described species of *Mico*. We therefore describe the marmosets from the northern Tapajós–Jamanxim interfluve as a new species of *Mico*.

Order Primates Linnaeus, 1758
Family Callitrichidae Gray, 1821
Genus *Mico* Lesson, 1840

***Mico munduruku* sp. n. R Costa-Araújo, IP Farias & T Hrbek, 2019**
urn:lsid:zoobank.org:act:FC41A13D-D365-4403-AEE8-8111C7446D68

**Holotype.** MPEG 45560, tissue CTGA-M 5898, field number RCA 24, adult male, stuffed skin, skull and skeleton, collected by Rodrigo Costa Araújo on October 10, 2015 on the right margin of the mouth of Crepori River, at Boca do Crepori community (05°46′55″S, 57°15′14″W), Itaituba municipality, Pará State, Brazil.

**Paratopotype.** MPEG 45559, tissue CTGA-M 5897, field number RCA 23, adult female, stuffed skin, skull and skeleton, collected by Rodrigo Costa Araújo on 10 October 2015 on the right margin of the mouth of Crepori River, at Boca do Crepori community (05°46′55″S, 57°15′14″W), Itaituba municipality, Pará State, Brazil.

**Paratypes.** MPEG 45622, adult female, field number JK 003, stuffed skin, tissue, collected by Arlindo-Jr. on 20 January 2013 on the right margin of the lower Crepori River (05°45′59″S; 57°17′14″W); INPA 7285, tissue CTGA-M 5910, field number RCA 36, adult female, stuffed skin, skull, skeleton, INPA 7284, tissue CTGA-M 5909, field number RCA 35, adult female in fluid, and INPA 7382, tissue CTGA-M 5911, field number RCA 37, adult female in fluid, all three collected by Rodrigo Costa Araújo on October 20, 2015 on the left margin of Jamanxim River at Jardim do Ouro community (06°15′11″S, 55°46′49″W), Itaituba municipality, Pará State, Brazil.

**Type locality.** Boca do Crepori community (05°46′55″S, 57°15′14″W), right margin of the mouth of the Crepori River, Itaituba municipality, Pará State, Brazil.

**Diagnosis.** The new species is unambiguously diagnosable from all other species of *Mico* by the possession of a white tail, feet and hands, white forearms with a beige-yellowish spot on the elbow, and beige-yellowish saddle.

**Etymology.** The specific epithet is a noun in apposition and honors the Munduruku Amerindians of the Tapajós–Jamanxim interfluve.

**Suggested vernacular names.** “sagui-dos-Munduruku” (Portuguese), “Munduruku marmoset” (English).

**Geographic Distribution.** *Mico munduruku* sp. n. is endemic to the Amazonian forest of the southwest of Pará State, Brazil, occurring from the left margin of the Jamanxim River, below the mouth of Novo River, possibly up to the right margin of the upper Tapajós River, below the mouth of Cururú River.

**Habitat.** Lowland primary and secondary *terra firme* forests.

**Description of the holotype.** Hair on face are short, sparse and white, around but not between the eyes, in circumbucal area, rhinarium and sides of the face; eumelanic and white vibrissae in the supraorbital region, rhinarium and following the zygomatic bone. Face tegument is vivid red overall and almost completely exposed, but albinotic around the nasal openings and on the inferior lip at the area of contact with upper canines; circumbucal tegument is pale yellow, extending as a narrow line through the center of the rhinarium; no eumelanic patch on face tegument; dark brown eyes. The hair on ear are white, short, and scarce, located on both surfaces of ear auditory, allowing the complete exposition of the pinnae; ear tufts absent; ear tegument vivid red as in the face. White hair on the sides of the head, longer than in face but shorter as to not cover pinnae; white hair on the head, crown undistinguished; the forehead has a lower density of white hair, partially allowing the exposition of a yellowish tegument. White mantle. Dorsal forearms are white with a beige-yellowish spot on the elbow, white hair on hands; ventral forelimbs are cream and chest is white. Saddle is beige-yellowish and laterally grades to a cream color nearby and on the belly. Hair on frontal and ventral regions of hind limbs are cream and grade to dark yellow toward the posterior region; dorsal hind limb and rump hair dark yellow; white hair on feet. White tail; only on the ventral and lateral surfaces of tail insertion hair are dark yellow. Ventral surface of hands and feet unpigmented; claw-like nails as in other marmoset species, curved dorsoventrally in all digits except the big toe, which bears a flat nail.

**Variation of the paratypes.** The pattern of pelage pigmentation is very similar in all type specimens and in the specimens observed in the field, with only slight tonal variation in the general appearance of colors in some chromogenetic fields. The saddle pigmentation varies in and between type specimens from a plain beige to beige-yellowish, and the rump and the dorsal underparts vary from dark yellow to plain yellow. The mantle can be less discrete, either by showing a narrow line of cream hair that cross the white mantle over the dorsal spine, or by showing cream hair in a more diffuse fashion in parts of the mantle.

**Comparisons with closely related species.** In contrast to *M. munduruku* sp. n., *M. leucippe* has an overall cream pelage, but the hands, feet and tail are golden; *M. emiliae* is overall grayish brown, with black feet, hands, tail, and crown. The tail of *M. intermedius* is cream with randomly dispersed black hair, more frequently in the proximal and in the distal portions; in addition, the posterior half of the saddle of *M. intermedius* bears black and cream hair, and the crown is a pale brown stripe. *M. argentatus* pelage is overall silvery, but deep brown to black on tail, and dark gray on feet and hands.

The electronic version of this article in portable document format will represent a published work according to the International Commission on Zoological Nomenclature (ICZN), and hence the new name contained in the electronic version is effectively published under that Code from the electronic edition alone. This published work and the nomenclatural acts it contains have been registered in ZooBank, the online registration system for the ICZN. The ZooBank Life Science Identifiers (LSIDs) can be resolved and the associated information viewed through any standard web browser by appending the LSID to the prefix http://zoobank.org/. The LSID for this publication is: urn:lsid:zoobank.org:pub:4F59F1BA-D6F0-4027-A762-FF96C23C5B0B. The online version of this work is archived and available from the following digital repositories: PeerJ, PubMed Central, and CLOCKSS.

## Discussion

Here, we demonstrate how an insight into the patterns of diversity and distribution of Amazonian marmosets lead to the discovery of a new species of *Mico* in the field. The Tapajós–Jamanxim interfluve in southern Amazonia is an area of ∼120,000 km^2^—nearly the size of England (130,000 km^2^) or four times the area of Belgium (30,000 km^2^)—embedded in the center of distribution of the genus *Mico*. However, no marmosets were ever collected in this region, and just a handful of primate and mammal surveys have been conducted, most of these very recently (Pimenta & Silva Júnior, 2005; Fialho, 2010; Ravetta, 2015; Buss et al., 2017). Therefore it is not entirely surprising that when we carried out marmoset-specific surveys in the Tapajós–Jamanxim interfluve we detected specimens with a distinct pelage pigmentation pattern in the northern portion of this interfluve.

After conducting field work across southern Amazonia to collect new distribution data, samples and voucher specimens, visiting museum collections to examine specimens and gather data on pelage pigmentation characters, tissue samples and distribution records, and reviewing the literature, we generated new information on pelage pigmentation patterns, phylogenomic relationships and geographic distribution of marmosets. The analyzes presented here, conducted under a criteria-driven integrative taxonomy framework (Schlick-Steiner et al., 2010; Padial et al., 2010), support the hypothesis that *M. munduruku* sp. n. represents a group of individuals distinctive in pelage pigmentation patterns that share a common and exclusive ancestry and that interbreed in an area largely isolated from closely related species by rivers. In this sense, *M. munduruku* sp. n. adheres to the biological and the phylogenetic species concepts. This is the first marmoset species to be described through an explicit hypothesis test and the adoption of objective criteria for taxonomic decision making.

The two phylogenetic reconstruction methods used here to infer the evolutionary relationships of species of *Mico* resulted in phylogenomic trees of identical topology and comparable support values for lineages (wherein the species are terminals). The Bayesian tree is strongly supported (≥0.99 posterior probabilities) at all nodes, as is the Maximum Likelihood tree (≥70% bootstrap support) (see Hillis & Bull, 1993) in all nodes except the *M. argentatus* clade and the polyphyletic group comprising *M. emiliae* and *M. leucippe*. These results support (i) the hypothesis of four lineages in the genus *Mico*—although this must be further investigated with the inclusion of molecular data from all the taxa of the genus—and (ii) the monophyly of *M. munduruku* sp. n., *M. intermedius*, and *M. rondoni. M. munduruku* sp. n. is strongly supported in all phylogenetic analyses, underpinning the hypothesis that these specimens share a common evolutionary history, which is reflected also in the distinctiveness of their pattern of pelage pigmentation.

Assessment of phylogenetic support via bootstrap and posterior probabilities is fundamentally different, bootstrap being a non-parametric and posterior probabilities a parametric measure. Hence the differences in support values (Huelsenbeck et al., 2002). However, Hillis & Bull (1993) have shown that bootstrap values >0.72 are equivalent to posterior probability of 0.95. Furthermore, it has also been shown that Bayesian analyses also tend to overestimate support of weaker nodes (Huelsenbeck et al., 2002) as would appear to be the case of *M. emiliae*, *M. leucippe*, and *M. argentatus*.

These phylogenetic relationships must be further explored with the inclusion of ddRAD sequences from more individuals of each species to test this pattern with more data, and to clarify potential underlying processes at population level. Preliminarily, we suggest that the low bootstrap support for the *M. argentatus* clade might be reflecting introgression events subsequent to secondary contact with *M. leucippe* in the Tapajós–Xingu interfluve, since the Bayesian tree supports the monophyletic origin of this taxon. The polyphyly of *M. emiliae* and *M. leucippe* may also be a reflection of introgression following a secondary contact, since there are no physical barriers to gene flow in between the two taxa; or the polyphyly might have been maintained by recurrent gene flow during speciation, or it simply reflects incomplete lineage sorting.

The southern and western portions of the geographic distribution of *M. munduruku* sp. n. need additional field surveys to be precisely defined. We confirmed the occurrence of *M. munduruku* sp. n. on both margins of the Crepori River based on interviews of multiple residents at three distinct localities along the course of the Crepori River. Based on landscape features and distribution records, we assume the Crepori River does not represent a physical barrier to dispersal of *M. munduruku* sp. n.. Better assessment of the geographic distribution of *M. leucippe* is also contingent on more field surveys since there are only two records of this species south of Novo and Cururú Rivers, in the southern Tapajós–Jamanxim interfluve.

Based on current knowledge, *M. munduruku* sp. n. is endemic to an area of approximately 55,000 km^2^ in the northern portion of Tapajós–Jamanxim interfluve, southwestern Pará State, Brazil. This area has suffered extensive environmental damages due to illegal logging and agricultural expansion—this is happening even within federal conservation units and protected indigenous lands (Printes, 2017). This region is one of the main fronts of forest destruction within the Arc of deforestation, a region infamously characterized by fast, intense and disordered conversion of forests to pastoral and agricultural land and human settlements. In addition, there are four hydroelectric plants in the process of implementation in this region that will directly impact the habitat of *M. munduruku* sp. n.. Thus, just as we have discovered this species, we already need to be concerned about its survival. *M. munduruku* sp. n. already faces the above mentioned threats, and these threats will only increase as hydroelectric plants and complementary infrastructure schemes as roads and transmission lines come to completion, inducing more intense settlement and forest reduction in the region. For these reasons, further research is urgently needed to better delimit the species’ range and to estimate population size and density, allowing us to assess its conservation status and, at least, mitigate some of the projected impacts on its populations.

## Conclusions

Our knowledge on species diversity and distribution in genus *Mico* is now slightly less incomplete and inaccurate with the description of *M. munduruku* sp. n., discovered during field expeditions to a distributional lacuna of Amazonian marmosets. The description of *M. munduruku* sp. n. raises the total number of species of *Mico* to 15, considering all taxa listed by Rylands & Mittermeier (2013) as valid. Nonetheless, further research is needed to clarify the phylogenetic relationships and the taxonomic status of Amazonian marmoset taxa—as here exemplified by the polyphyly of *M. emiliae* and *M. leucippe*, and by the previous opinion on the synonymy of *M. manicorensis* and *M. marcai* (Garbino, 2015).

The existence of large, previously unsurveyed areas for marmosets in southern Amazonia, and the description of *M. munduruku* sp. n. from one of these areas, highlights that: (i) Amazonian marmosets represent one of the least-studied groups of Neotropical primates, and thus (ii) further field-based and integrative studies are needed to clarify species diversity and distribution of this group (see also Ferrari, 2009). Our study also highlights the need for the collection of field records and biological material, without which our field discovery and integrative taxonomic approach would not be possible. Another issue is the need for marmoset-specific surveys to detect these monkeys, whether in dense forests or secondary vegetation. In fact, the current lack of data on Amazonian marmosets is due not only to lack of field surveys, but also to their low detectability (small size and reclusive behavior). Marmosets are unlikely to be recorded in general surveys of primates or mammals or, when recorded, they are often detected only by their characteristic long-call vocalizations or as fleeting observations during rapid surveys which can lead to species misidentification.

Considering the scarcity of information on diversity and distribution of *Mico* species and the intensive habitat reduction in the Arc of deforestation, which entirely encompasses their ranges, more studies are urgently needed in order to generate the information necessary for science driven conservation initiatives. Without a reliable basis on species diversity and distribution, conservation efforts on this group of monkeys are largely ineffective. At this moment, *M. munduruku* sp. n. should be considered as Data Deficient under the IUCN criteria for assessment of species conservation status.

## Supplemental Information

10.7717/peerj.7019/supp-1 Supplemental Information 1 Schematic representation of a dorsal view of a marmoset skin and the pelage pigmentation characters (chromogenetic fields) as here examined. Image credit: Rodrigo C. Araújo.

10.7717/peerj.7019/supp-2 Supplemental Information 2 One of the four lineages of the genus *Mico*. Left to right: *Mico munduruku* sp. n., *M. leucippe*, *M. emiliae*, *M. argentatus*, *M. rondoni*, *M. intermedius*. Image credit: Stephen Nash.

10.7717/peerj.7019/supp-3 Supplemental Information 3 Maximum Likelihood phylogeny of the genus *Mico*. Grey-scale bars represent the main species lineages in genus *Mico*, black bar represent the outgroups. Bootstrap support values are given above nodes. Image credit: Stephen Nash.

10.7717/peerj.7019/supp-4 Supplemental Information 4 Framework adopted in this study for taxonomic assessment and decision making, based on Schlick-Steiner *et al.* (2010).

10.7717/peerj.7019/supp-5 Supplemental Information 5 Prepared skins and specimens in fluid examined (N=521) in museums for collection of data on pelage pigmentation characters. The type specimens are indicated with bold letters and the specimens obtained for this study are indicated with asterisk (^*^). Field Museum of Natural History (FMNH), Instituto Nacional de Pesquisas da Amazônia (INPA), Museu Paraense Emilio Goeldi (MPEG), Museu de Zoologia da Universidade de São Paulo (MZUSP), Natural History Museum of London (NHM), Naturhistorisches Museum Wien (NMW), and Universidade Federal de Rondônia (UNIR).

10.7717/peerj.7019/supp-6 Supplemental Information 6 Specimens stored in mammalian collections sampled to generate phylogenomic data (ddRAD sequences) for inference of evolutionary relationships in *Mico*. Specimens obtained in this study are indicated with asterisk (^*^). Coleção de Tecidos de Genética Animal (CTGA) of the Universidade Federal do Amazonas, Instituto Mamirauá de Desenvolvimento Sustentável (IDSM), Instituto Nacional de Pesquisas da Amazônia (INPA), Museu Paraense Emilio Goeldi (MPEG), Universidade Federal do Mato Grosso (UFMT), and Universidade Federal de Rondônia (UNIR).

## Additional Information and Declarations

### Field Study Permissions

The following information was supplied relating to field study approvals (i.e., approving body and any reference numbers): All samples were collected with the permission of relevant country organizations. Collections of biological material were conducted under the licenses ICMBio/IBAMA 50416-6 of Rodrigo Costa Araújo.

### Data Availability

The following information was supplied regarding the deposition of DNA sequences: The sequence data are available at https://github.com/legalLab/publications and at GenBank: PRJNA552061.

### New Species Registration

The following information was supplied regarding the registration of a newly described species: Publication LSID: urn:lsid:zoobank.org:pub:4F59F1BA-D6F0-4027-A762-FF96C23C5B0B. Species LSID: *Mico munduruku* Costa-Araújo, Farias & Hrbek in Costa-Araújo, Melo, Canale, Hernández-Rangel, Messias, Rossi, Silva, da Silva, Nash, Boubli, Farias & Hrbek, 2019. urn:lsid:zoobank.org:act:FC41A13D-D365-4403-AEE8-8111C7446D68.
