# Supplementary figures and images for: The Munduruku marmoset: a new monkey species from southern Amazonia
Source: PeerJ. 2019 Jul 25;7:e7019. doi: 10.7717/peerj.7019 (PMC6661146; doi:10.7717/peerj.7019)

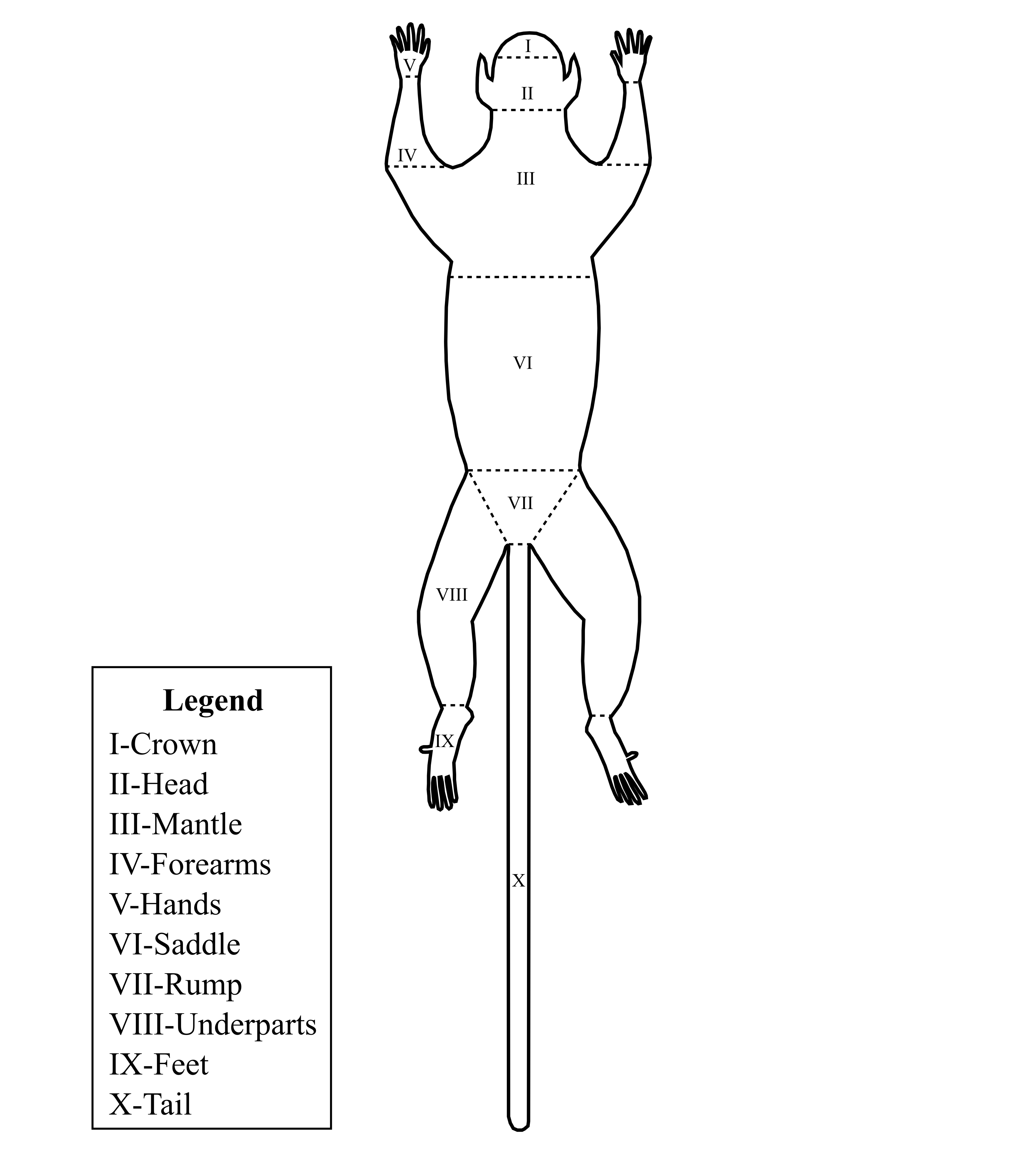

Supplement: Supplemental Information 1 — Image credit: Rodrigo C. Araújo. [file peerj-07-7019-s001.png]

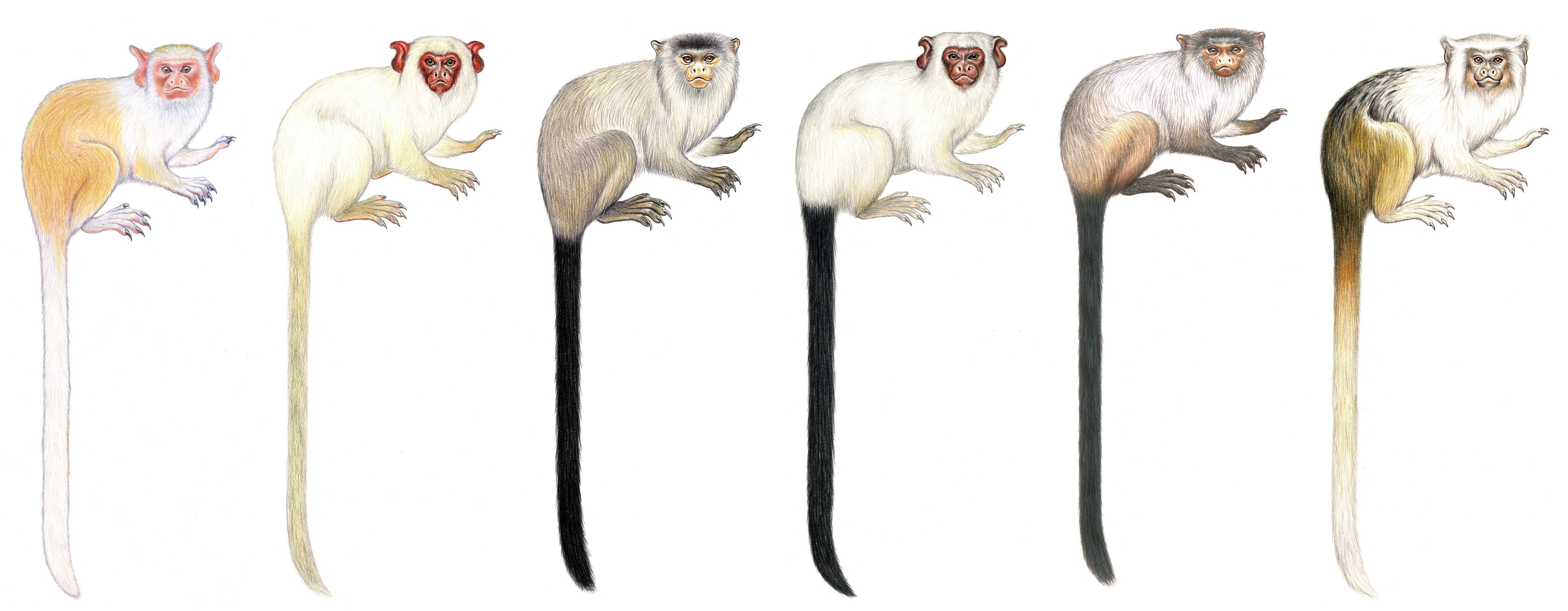

Supplement: Supplemental Information 2 — Left to right: Mico munduruku sp. n., M. leucippe, M. emiliae, M. argentatus, M. rondoni, M. intermedius. Image credit: Stephen Nash. [file peerj-07-7019-s002.png]

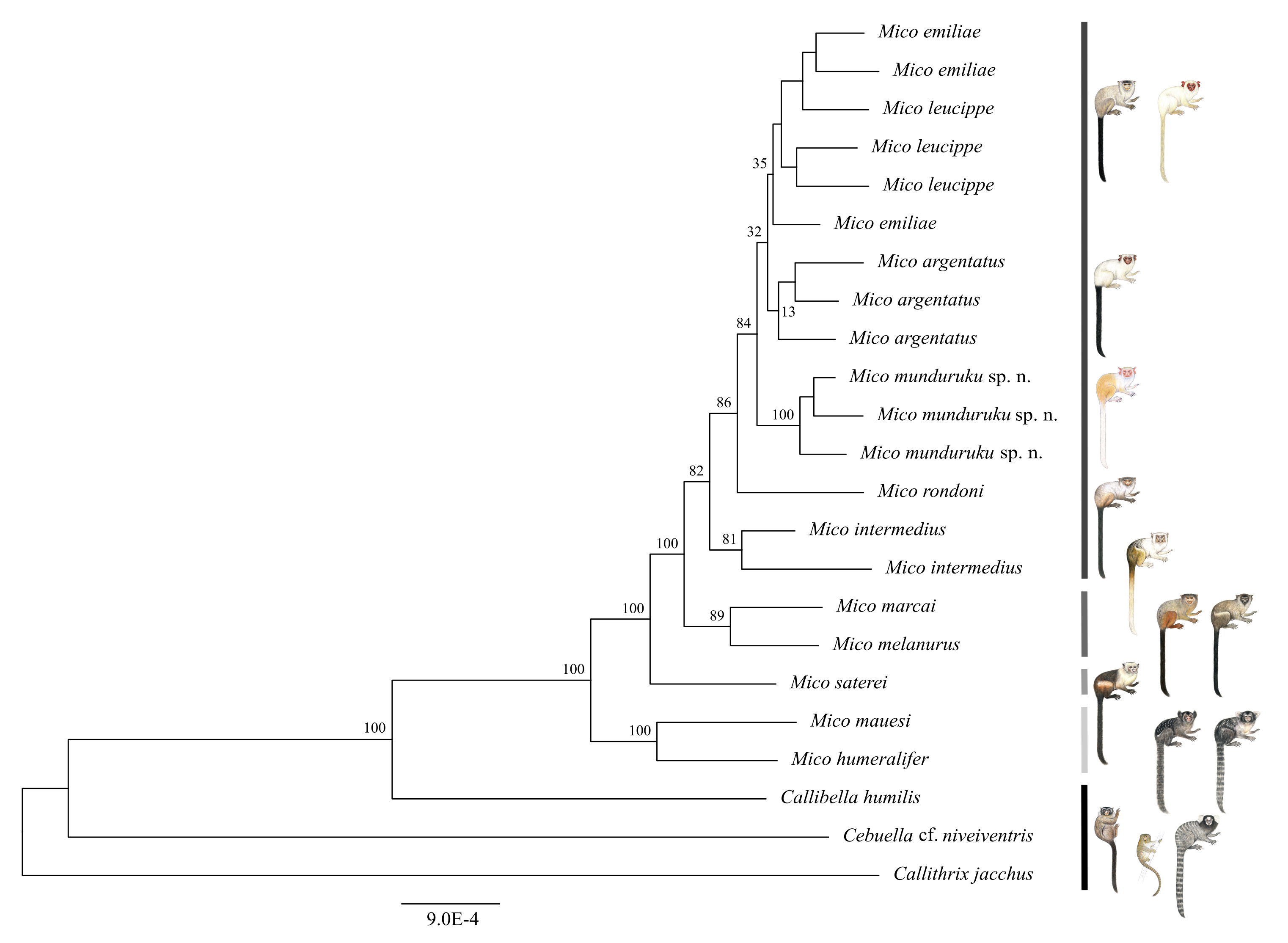

Supplement: Supplemental Information 3 — Grey-scale bars represent the main species lineages in genus Mico, black bar represent the outgroups. Bootstrap support values are given above nodes. Image credit: Stephen Nash. [file peerj-07-7019-s003.png]
