# Supplementary material for: The Munduruku marmoset: a new monkey species from southern Amazonia
Source: PeerJ. 2019 Jul 25;7:e7019. doi: 10.7717/peerj.7019 (PMC6661146; doi:10.7717/peerj.7019)
Supplement: Supplemental Information 4 [file peerj-07-7019-s004.pdf]

Table S1. Framework adopted in this study for taxonomic assessment and decision making, based on Schlick-Steiner *et al.* (2010).

**Sources of information**	**Criteria**	**Single source**	**Integrated sources**
**Pelage pigmentation data**<br>Pelage pigmentation pattern	Phenotypic distinctness	H0 – H1	
**Phylogenomic data**<br>Genome-wide sampling of nuclear DNA	Monophyly	H0 – H1	H0 – H1
**Distribution data**<br>Localities of occurrence	Allopatry/ Parapatry	H0 – H1	
