# Supplementary material for: The Munduruku marmoset: a new monkey species from southern Amazonia
Source: PeerJ. 2019 Jul 25;7:e7019. doi: 10.7717/peerj.7019 (PMC6661146; doi:10.7717/peerj.7019)
Supplement: Supplemental Information 5 — The type specimens are indicated with bold letters and the specimens obtained for this study are indicated with asterisk (*). Field Museum of Natural History (FMNH), Instituto Nacional de Pesquisas da Amazônia (INPA), Museu Paraense Emilio Goeldi (MPEG), Museu de Zoologia da Universidade de São Paulo (MZUSP), Natural History Museum of London (NHM), Naturhistorisches Museum Wien (NMW), and Universidade Federal de Rondônia (UNIR). [file peerj-07-7019-s005.pdf]

Taxon	Specimen code
Mico acariensis (n=6)	INPA: **3931**; MPEG: 45578*, 45579*, 45580*, 45581*, 45582*.
Mico argentatus (n=151)	FMNH: 19499, 19533, 50839, 50840, 50841, 92177, 92178, 92179, 92180; INPA: 2473; MPEG: 29, 38, 151, 154, 156, 157, 162, 163, 164, 165, 165, 166, 269, 328, 336, 6878, 8951, 8952, 8982, 8983, 8984, 8985, 8986, 8987, 9200, 9201, 9202, 9203, 9204, 9205, 9206, 9207, 9208, 9212, 10021, 10022, 10023, 10024, 10025, 10026, 21373, 21374, 21375, 21376, 21377, 21378, 21379, 21380, 21381, 21382, 21383, 21384, 21385, 21386, 21387, 21388, 21389, 21390, 21391, 21392, 21393, 21394, 21414, 21630, 21631, 21632, 21633, 21634, 21635, 21636, 21637, 22922, 22923, 22924, 22925, 22926, 22927, 22928, 22929, 23156, 23157, 23158, 45605*, 45606*, 45607*, 45608*, 45609*; MZUSP: 3588, 3589, 3591, 3593, 3594, 3595, 3596, 4313, 4829, 4833, 4840, 4865, 4899, 4900, 4901, 4902, 4903, 4904, 4905, 4906, 4907, 4908, 4909, 4910, 4911, 4913, 4914, 4915, 4916, 4918, 4940, 4959, 4964, 4965, 4966, 4967, 4968, 4969, 4970, 4971, 4972, 4973, 4974, 4975, 5007, 5026, 6633, 11272, 11307, 11308, 11367, 11407, 11307, 11308.
Mico chrysoleucos (n=60)	FMNH: 50821, 50822, 50823, 50825, 50826, 50827, 50828, 50829, 50830, 50831, 50832, 50833, 50834; INPA: 4110, 4038, 4039, 7388*, 7389*, 7390*; MPEG: 237, 544, 23064, 45576*, 45577*, 45587*, 45590*, 45591*, 45610*; MZUSP: 4210, 4211, 4884, 4885, 4886, 4887, 4888, 4890, 4892, 4893, 4894, 4976, 5006, 5008, 5009, 5018, 5019, 5020, 5022, 5028, 5029, 5030, 11246, 11409,

	11410, 11411, 13466, 13467; NMW: **ST 970**, **B 3455**, **B 3456**, **B 3457**.
Mico emiliae (n=10)	INPA: 7287*, 7288*, 7289*, 7290*; MPEG: **170**, 37807, 37808, 37809, 45566*; MZUSP: 35106.
Mico humeralifer (n=124)	FMNH: 19508, 92165, 92166, 92167, 92168, 92169, 92170, 92171, 92172, 92173; INPA: 4111, 4083, 7385*, 7386*, 7282*; MPEG: 30, 172, 173, 174, 175, 178, 265, 266, 267, 268, 337, 1384, 9213, 21397, 21398, 21399, 21400, 21401, 21402, 21403, 21404, 21405, 21406, 21407, 21408, 21409, 21410, 21411, 21412, 38387, 38504, 38505, 39474, 39475, 40984, 44282, 44283, 5904*, 5905*; MZUSP: 3577, 3578, 3579, 3580, 3582, 3584, 3585, 3586, 4927, 10095, 10096, 10097, 10098, 10099, 11249, 11250, 11251, 11252, 11253, 11254, 11255, 11256, 11257, 11258, 11259, 11260, 11261, 11262, 11263, 11264, 11265, 11266, 11268, 11269, 11270, 11271, 11294, 11297, 11298, 11299, 11300, 11301, 11302, 11303, 11304, 11306, 11309, 11310, 11312, 11332, 11333, 11347, 11348, 11349, 11350, 11351, 11356, 11357, 11358, 11360, 11393, 11396, 11397, 11398, 11399, 11400, 11401, 11412, 11413, 18866.
Mico intermedius (n=9)	MPEG: **8156**, **12599**, 23065, 45585*, 45592*, 45594*, 45595*, 45598*, 45600*.
Mico leucippe (n=29)	FMNH: 92174, 92175, 92176; MPEG: 43661, 45563*, 45564*, 45565*, 45567*, 45568*, 45569*; MZUSP: 3598, 9964, 9965, 10093, 10094, 11248, 11279, 11280, 11281, 11291, 11295, 11296, 11305, 11311, 11361, 11394, 11402, 11403. NHM: **9.3.9.2**
Mico manicorensis (n=1)	INPA: **2511**.

Mico marcai (n=2)	MPEG: 42807, 45641.
Mico mauesi (n=11)	INPA: 4105, 4106, 4107, 4108, 4109, 4082; MPEG: **22177**, 23962, 23963, 23964; MZUSP: 29027.
Mico melanurus (n=36)	FMNH: 26730, 44859, 51888; INPA: 7296*, 7283*, 7383*; MPEG: 15266, 15267, 21395, 21396, 45571*; MZUSP: 3367, 3368, 3369, 3370, 3376, 4263, 4264, 4265, 4266, 6327, 6328, 6329, 6330, 6332, 6333, 24734; NMW: ST 1578, B 3447, B 3448, B 3449, B 3451, B 3758; UNIR: 192, 346, 356.
Mico munduruku sp. n. (n=6)	INPA: **7284***, **7285***, **7382***; MPEG: **45559***, **45560***, **45622**.
Mico nigriceps (n=17)	MPEG: **21996**, **21997**, **21998**, **21999**, 22955, 22956, 22957, 22958, 22959, 22960, 22961, 22962, 45614*, 45615*, 45616*, 45617*, 45618*.
Mico rondoni (n=45)	MPEG: 21365, **21366**, **21367**, **21646**, **21647**, 21648, 21649, 21650, 21651, 21652, 21653, 21654, 21655, 21656, 21657, 21658, **21659**, **21660**, 21885, **21886**, 21887, 21888, 21889, 21891, 21892, **21893**, **21894**, 21895, **21896**, **21897**, 21898, 28691, 45620*; MZUSP: 20142; UNIR: 15, 26, 48, 78, 159, 162, 165, 396, 556, 639, 158.
Mico saterei (n=14)	INPA: 4040, 4101, 4102, 4103, 4104, 4082, 5672; MPEG: **23955**, **23956**, **23957**, **23958**, **23959**, **23960**, **23961**.
