# Supplementary material for: The Munduruku marmoset: a new monkey species from southern Amazonia
Source: PeerJ. 2019 Jul 25;7:e7019. doi: 10.7717/peerj.7019 (PMC6661146; doi:10.7717/peerj.7019)
Supplement: Supplemental Information 6 — Specimens obtained in this study are indicated with asterisk (*). Coleção de Tecidos de Genética Animal (CTGA) of the Universidade Federal do Amazonas, Instituto Mamirauá de Desenvolvimento Sustentável (IDSM), Instituto Nacional de Pesquisas da Amazônia (INPA), Museu Paraense Emilio Goeldi (MPEG), Universidade Federal do Mato Grosso (UFMT), and Universidade Federal de Rondônia (UNIR). [file peerj-07-7019-s006.pdf]

Taxon	Specimen code
Callibella humilis	**INPA** 4090.
Cebuella cf. *niveiventris*	**CTGA-M** 170.
Mico argentatus	**CTGA-M** 434, 6015, 6016.
Mico emiliae	**INPA** 7287*, 7289*; **MPEG** 45566*.
Mico humeralifer	**INPA** 7282*.
Mico intermedius	**IDSM** 792; **UFMT** 4705.
Mico leucippe	**MPEG** 45568*, 45569*; **CTGA-M** 5912*.
Mico marcai	**MPEG** 45641.
Mico mauesi	**CTGA-M** 5881*.
Mico melanurus	**INPA** 7383*.
Mico munduruku sp. n.	**INPA** 7284*, 7382*; **MPEG** 45559*.
Mico rondoni	**UNIR** 556.
Mico saterei	**INPA** 4104.
